# Cereblon: A Protein Crucial to the Multiple Functions of Immunomodulatory Drugs as well as Cell Metabolism and Disease Generation

**DOI:** 10.1155/2017/9130608

**Published:** 2017-08-15

**Authors:** Qinglin Shi, Lijuan Chen

**Affiliations:** Department of Hematology, The First Affiliated Hospital of Nanjing Medical University/Jiangsu Province Hospital, 300 Guangzhou Road, Nanjing 210029, China

## Abstract

It is well known that cereblon is a key protein in autosomal recessive nonsyndromic mental retardation. Studies have reported that it has an intermediary role in helping immunomodulatory drugs perform their immunomodulatory and tumoricidal effects. In addition, cereblon also regulates the expression, assembly, and activities of other special proteins related to cell proliferation and metabolism, resulting in the occurrence and development of metabolic diseases. This review details the multiple functions of cereblon and the underlying mechanisms. We also put forward some unsolved problems, including the intrinsic mechanism of cereblon function and the possible regulatory mechanisms of its expression.

## 1. Introduction

The gene that encodes cereblon, *CRBN*, was first identified by Higgins et al. while studying genes that were related to memory and learning. Their team found a nonsense mutation (R419X) in a newly discovered gene located on 3p26.2 in an ethnic group with a mild type of nonsyndromic mental retardation. The gene was assigned the name *CRBN* (cereblon, NM_016302) based on its supposed role in the development of cerebral tissues and because its expression in the hippocampus among other areas is associated with memory and learning processes [1]. Although there are numerous causes of mental retardation, the stop codon of the CRBN gene is thought to be the major factor [2].

CRBN interacts with the DNA damage-binding protein-1 (DDB1), Cullin 4 (Cul4A or Cul4B), and regulator of Cullins 1 (RoC1) to form the functional E3 ubiquitin ligase complex. In this complex, CRBN functions as a substrate receptor of E3 ubiquitin ligase complex and targets proteins for proteolysis through a ubiquitin-proteasome pathway [2–4]. E3 ubiquitin ligase complex can achieve different effects by targeting different substrates.

In recent years, CRBN has been extensively studied because it is involved in many biological processes and is responsible for the multiple effects of immunomodulatory drugs (IMiDs). CRBN performs these functions generally under two circumstances: with and without IMiDs. CRBN expression in the cells can affect cell metabolism and cause disease in the absence of IMiDs. In addition, CRBN is also the target protein of IMiDs and enhances their effects when present. In this review, we will describe the various functions of CRBN and the underlying mechanisms involved.

## 2. CRBN Expression Affects Cell Metabolism and Disease Generation

CRBN, a 442-amino acid protein with multifunction, locates in the cytoplasm, nucleus, and peripheral membrane of the human brain and other tissues [5]. The diverse roles of CRBN on cell metabolism and disease genesis have been extensively studied.

### 2.1. CRBN and Cell Metabolism

**Figure 1:**
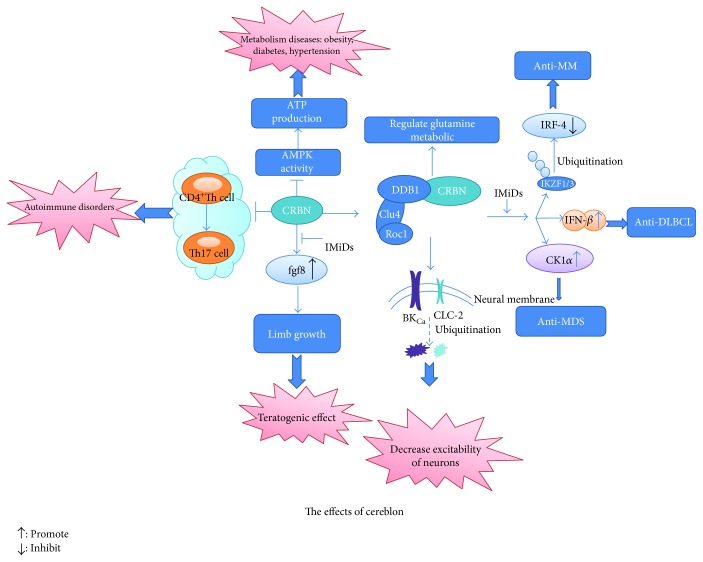
Schematic representation of CRBN-mediated function regulation. In the absence of immunomodulatory drugs (IMiDs), CRBN has an important role in the regulation of ion transport, modulation of AMP-activated protein kinase (AMPK) signaling, and metabolism in cell and whole tissues or organs. In the presence of IMiDs, CRBN is the target protein of IMiDs and helps exert their multiple functions.

CRBN has an important role in the regulation of ion transport, modulation of AMP-activated protein kinase (AMPK) signaling pathway, and metabolism in cell and whole tissues or organs (Figure 1) [1, 6, 7]. Intriguingly, CRBN also influences cell proliferation and apoptosis.

Large-conductance Ca^2+^-activated K^+^ (BK_Ca_) channels are ubiquitous in many tissues and are activated by membrane depolarization and high levels of intracellular calcium [8]. BK_Ca_ channels have an essential role in neuronal excitability. Evidence showed that CRBN might regulate the activity of BK_Ca_ channels by affecting their expression and assembly in the cell membrane [9]. BK_Ca_ channels are ubiquitinated in the endoplasmic reticulum by the E3 ubiquitin ligase complex, through direct interaction with CRBN. This process decreases the expression of BK_Ca_ channels in the neuronal membrane surface and retains them in the endoplasmic reticulum [4, 10]. When this control process is inhibited, BK_Ca_ channels in the cell membrane are enriched and the excitability of neurons is increased. These effects increase excitability of nerve cells and eventually increase the risk of epilepsy [8, 10]. In addition to BK_Ca_ channels, experiments showed that CRBN also interacts with the voltage-gated chloride channel-2 (ClC-2), which is ubiquitous in cell membranes and functions by regulating cell excitability in neurons [11, 12]. CRBN helps the E3 ubiquitin ligase complex target ion channels for ubiquitination and therefore maintains the ion balance and reduces the incidence of ion channel disease.

CRBN is a metabolic regulator that directly binds to AMPK and inhibits its function of increasing ATP production and decreasing ATP consumption. Its regulation of energy metabolism on a cellular level helps to control appetite, intake of nutrients, and the endocrine system, which are related to many physiological processes [13, 14]. AMPK regulates the metabolism of carbohydrates, liquid, proteins, and the energy balance of the whole body. The dysfunction of AMPK causes a wide spectrum of metabolic diseases such as obesity, diabetes, hypertension, and even cancer [13].

The CRBN protein also participates in the regulation of glutamine whose dysregulation is responsible for many diseases including cancer and other metabolic abnormalities [15, 16]. Glutamine synthetase (GS) is essential in the signaling pathway that regulates glutamine levels. Nguyen et al. reported that GS binds directly to CRBN, leading to GS ubiquitination by E3 ubiquitin ligase complex and the eventual decrease of glutamine. This process functions as a negative feedback mechanism under high glutamine concentrations [17, 18]. Likewise, the ubiquitination of GS will be reduced under low glutamine concentrations.

In addition to the regulation of key proteins and signal pathways related to cell metabolism, CRBN has effects on cell proliferation and apoptosis. CRBN knockdown in multiple myeloma cells showed significantly reduced CRBN expression and decreased cell viability [19]. In contrast, CRBN overexpression promotes cell proliferation.

### 2.2. CRBN and Disease Genesis

Many diseases, such as cardiovascular disease, obesity, and fatty liver, have been linked to the CRBN-mediated inactivation of AMPK. Several studies demonstrated that activated AMPK protected myocardial tissues, thus decreasing ischemia-reperfusion injury. The activity inhibition of AMPK by CRBN may cause cardiac diseases [20]. Furthermore, increased AMPK activity might protect cells from injury caused by high-fat induced disorders of lipometabolism and alcohol-induced accumulation of liquid in liver cells [13, 21, 22]. It was reported that CRBN-deficient mice were not susceptible to metabolic-related diseases (Figure 1) [23].

Recent studies also found that CRBN has a negative regulation role of CD4+ T cell activation. CRBN deficiency increased the activation of CD4+ T cells and enhanced IL-2 secretion, helping CD4+ T cells differentiate into Th17 cells [24]. Enhanced T cell activation can sometimes cause T-cell-mediated autoimmune disorders such as experimental autoimmune encephalomyelitis and delayed-type hypersensitivity reaction [25, 26]. These experiments reflect the regulatory role of CRBN in disease genesis.

Sawamura et al. reported, in 2015, that CRBN was recruited to aggresome and had a protective effect against extracellular stresses, such as ubiquitin-proteasome system (UPS) impairment and oxidative stress. Aggresomes are thought to be cytoprotective because they sequester toxic, aggregate proteins and eliminate them by autophagy. Actually, aggresomes which contain misfolded proteins can also be observed in many neuropsychiatric diseases such as Parkinson's disease and Schizophrenia. The team showed that CRBN plays a vital role in aggresome formation and cytoprotection against UPS impairment. The normal CRBN function is cytoprotective against UPS dysfunction-induced cell death and the defect may be of great importance to intellectual disability (ID) pathogenesis [27].

## 3. CRBN Is the Target Protein of IMiDs and Is Responsible for Their Multiple Functions

IMiDs, such as thalidomide, lenalidomide, and pomalidomide, are oral medications used for treating multiple myeloma (MM), deletion (5q) myelodysplastic syndrome (del(5q) MDS), chronic lymphocytic lymphoma (CLL), and activated B-cell-like subtype diffuse large B-cell lymphoma (ABC-DLBCL) (Figure 1). Thalidomide was first introduced in the late 1950s as a sedative for pregnant women to prevent morning sickness [28]. However, treatment with this drug caused serious side effects including limb deformity [28]. Because of this serious teratogenic effect, thalidomide was withdrawn from the market in 1960s [29]. Accumulating evidence indicated that CRBN was responsible for the teratogenic activities of thalidomide, until 2010, when a group of scientists proved that CRBN was the bona fide target of thalidomide [4]. Surprisingly, it was subsequently discovered that thalidomide possessed other activities including antiangiogenesis and anti-inflammatory effects. Based on the reports of thalidomide's effects against multiple myeloma, the Food and Drug Administration (FDA) approved the use of thalidomide for the treatment of the newly diagnosed MM patients in 1999 [29, 30]. Lenadilomide and pomalidomide were approved by FDA for MM treatment in 2006 and 2013, respectively. Those findings triggered increased researches in the cancer treatment field of IMiDs. The molecular mechanism of the antimyeloma effect of IMiDs has been studied over the last 20 years [31]. It was gradually discovered that the antitumor effect of IMiDs on multiple myeloma and other hematologic diseases is mediated by CRBN either through a ubiquitin-dependent or a ubiquitin-independent pathway. Furthermore, the response to IMiD therapy is also related to the expression of CRBN.

### 3.1. CRBN and the Teratogenic Effect of IMiDs

When it was discovered that CRBN directly interacted with thalidomide, researchers then investigated the role of CRBN *in vivo* [4]. Experiments on a zebrafish model finally verified the assumption that CRBN was responsible for the thalidomide teratogenic effect. In their experiments, thalidomide decreased the protein level of fibroblast growth factor 8 (fgf8), which is essential for limb growth [32]. Knockdown of zebrafish CRBN had the same effect as thalidomide treatment. Furthermore, the teratogenic effect induced by thalidomide was reversed by overexpression of wild-type CRBN. They also found that the overexpression of Y374A/W376A-mutated zebrafish CRBN, which cannot bind to thalidomide, had no obvious malformation of limb development. Therefore, CRBN was considered the direct target protein of thalidomide. Inhibition of CRBN activity by thalidomide downregulated fgf8. This is one of the mechanisms that caused the thalidomide teratogenic effect [4].

fgf8 can be upregulated by the CD147-MCT1 complex whose activity is promoted by CRBN through their combination. In the presence of thalidomide, the combination of CRBN and the CD147-MCT1 complex is weakened. Consequently, fgf8 expression is downregulated. This may be another mechanism of the teratogenic effect of IMiDs [33].

### 3.2. CRBN and the Antimyeloma Effect of IMiDs

The mechanism of the antimyeloma effect of thalidomide and other IMiDs remained unclear until researchers discovered the mechanism of the teratogenic effect of thalidomide and its direct combination with CRBN (Figure 1). Several researchers reported that Ikaros (IKZF1) and Aiolos (IKZF3) were direct substrates of E3 ubiquitin ligase complex. IKZF1 and IKZF3 are specific members of the B-cell transcription factors family and are critical for plasma cell development and proliferation [34]. After binding of IMiDs, E3 ubiquitin ligase complex changed its specificity and ubiquitinates and marks for degradation these two factors [34–36]. Interestingly, it was reported that a single amino acid substitution of IKZF3 decreased the inhibition of cell proliferation and conferred resistance to degradation induced by IMiDs [37]. These results suggested that the repression and degradation of IKZF1 and IKZF3 is potentially involved in the mechanism of IMiDs against multiple myeloma.

The degradation of IKZF1 and IKZF3 induces cytotoxicity in myeloma cells because they are critical factors for B-cell differentiation. Nevertheless, some investigators further studied the targets of these two factors to identify the most direct factors leading to this antitumor effect. Their results showed that the knockdown of IKZF1 and IKZF3 by sh-RNA contributed to the suppression of IRF4 levels and the increase of interleukin-2 (IL-2) levels, which affected the survival of MM cells. Similar results were observed under lenalidomide treatment [34].

The transcription factor IRF4, identified as an essential factor for myeloma cell survival, is involved in the activity of lenalidomide to treat multiple myeloma [38, 39]. The inhibition of IRF4 was toxic to myeloma cell lines. The direct targets of IRF4 include several key regulators that have a great impact on cell proliferation and survival, such as Myc, CDK6, and STAG2 [30, 38]. IRF4 regulates cell metabolism, cell cycle progression, cell death, and plasma cell differentiation via these direct targets [38].

It was reported previously that IL-2 inhibits tumor formation suggesting that the IL-2-mediated suppression of tumors might be a new approach for treating myeloma or other malignancies. IKZF1 and IKZF3 are transcriptional suppressors of the *IL-2* gene whose expression product can regulate T cell function [40, 41]. Therefore, lenalidomide-induced IL-2 production in T cells is caused by rescuing inhibition of IL-2 whose expression is repressed by IKZF1 and IKZF3 [40, 42].

In addition to the Ikaros family members, many other interesting proteins participating in important physiological processes also bind to CRBN and are regulated by IMiDs in a ubiquitin-dependent mechanism [37, 43].

In addition to the ubiquitin-dependent pathway mentioned above, a recent study reported a ubiquitin-independent pathway through which CRBN mediates the antimyeloma effect of IMiDs. CRBN promotes the activation of the CD147-MCT1 complex, which is upregulated in MM cells. CD147 enhances angiogenesis, cell proliferation, cell survival, and tumor aggressiveness, which can be observed in many kinds of malignances [44]. CD147 and MCT1 form a complex that functions in the cellular metabolism of tumor cells [45]. IMiDs compete with CRBN to combine with the CD147-MCT1 complex to weaken the CRBN and CD147-MCT1 complex, thus inhibiting tumor growth [33]. The suppression of CD147-MCT1 complex activity also decreases the aggressiveness of B cell neoplasms.

Recently, Xu et al. identified that argonaute 2 (AGO2), as a CRBN binding partner, plays an important role in regulating angiogenesis and MM cell survival. Treating the IMiD-sensitive MM cells with lenalidomide induced the steady-state levels of CRBN which were significantly increased whereas the levels of AGO2 were significantly decreased. It has been reported that AGO2 plays a pivotal role in microRNA (miRNA) maturation, stability, and function. Under the treatment of IMiDs, the steady-state levels of AGO2 and miRNA were significantly downregulated and ultimately inhibited angiogenesis and cell growth [46].

### 3.3. CRBN and Its Downstream Protein Expression Level Affect the Response Rate of IMiDs

Researchers started to explore whether CRBN protein level could be used to guide the rational clinical use of IMiDs. A study enrolled 107 MM patients to assess the expression level of CRBN protein by immunohistochemical staining. Among these patients, 60 were relapsed and/or refractory MM patients who had received lenalidomide and dexamethasone (LD) as their salvage treatment, 45 were newly diagnosed MM patients who had received thalidomide and dexamethasone (TD) as their induction therapy, and 22 were newly diagnosed patients who had melphalan, bortezomib, and prednisolone (MVP) as their induction therapy. Results suggested that higher CRBN protein level was associated with superior treatment response to IMiD-based therapy instead of the regimen without IMiDs [36]. Another study compared the changes of CRBN expression level pre- and post-lenalidomide therapy in nine lenalidomide-resistant patients. They were surprised to find that the CRBN expression level was significantly reduced at the time of drug resistance [19]. Similar studies have shown that CRBN is a unique biomarker for IMiD sensitivity and that high CRBN expression is an independent factor related to a better prognosis in MM patients treated with IMiDs [36, 47–49]. Some studies showed that the downstream of CRBN, such as IKZF1/3, may also affect the prognosis and the overall survival and progression-free survival of MM patients [36, 50, 51]. However, there is still much controversy in the relationship between CRBN-IKZF1/3-IRF4 expression level and prognosis in MM patients.

In line with the analysis of clinical data, the sensitivity to IMiDs is strongly related to CRBN protein level on a cellular level. Zhu et al. reported that the therapeutic effect of IMiDs was almost completely cancelled and IMiD-resistant cells formed if CRBN was knocked down [19]. Zhu et al. reported that *CRBN* knockdown caused MM cell lines to acquired resistance to lenalidomide compared with their counterparts. Subsequently, they did more tests with other antimyeloma drugs, such as pomalidomide, melphalan, dexamethasone, and bortezomib, and found that CRBN knockdown cells acquire resistance to pomalidomide but retained sensitivity to other drugs [19].

The same group compared the CRBN expression level of IMiDs primary resistance MM cell lines such as OCI-My5 and OPM1 with relatively sensitive cell lines such as MM1S and OPM2. Results showed that the sensitive cell lines expressed higher levels of CRBN protein and transcriptional levels than resistance cell lines [30]. Furthermore, the sensitivity of lenalidomide was increased by the upregulation of CRBN levels in the primary LEN-resistance cell lines [30]. Many other experiments also confirmed that acquired resistance to lenalidomide was accompanied by decreased CRBN. This evidence further demonstrated that CRBN is an independent factor that is predictive for the prognosis of MM patients using IMiD-based therapy [47, 52].

### 3.4. CRBN and the Therapeutic Effect of IMiDs in Other Hematologic Diseases

CRBN-associated substrates and downstream signaling vary in different cell types, which accounts for the multiple effects of IMiDs [30]. In addition to the antimyeloma effects, IMiDs are effective for the treatment of ABC-DLBCL, CLL, and deletion (5q) myelodysplastic syndrome (MDS). Although these therapeutic effects depend on CRBN, the pathways involved in cell proliferation and survival are different from those involved in the antimyeloma effect (Figure 1) [39, 53].

Lenalidomide-induced tumoricidal effects on ABC-DLBCL cells have gained much attention in recent years. CRBN is required for the toxic effect of lenalidomide in ABC-DLBCL. Knockdown of CRBN reduces the toxic effect of lenalidomide and affects its therapeutic ability on ABC-DLBCL. However, these effects can be reversed by the ectopic expression of CRBN [39]. Indeed, these therapeutic effects are achieved by enhancing interferon beta (IFN-*β*) production and inhibiting the activity of nuclear factor-*κ*B (NF-*κ*B) through the downregulation of IRF4 and SPi-B (an Ets family transcription factor) in a cereblon-dependent manner [54]. CRBN has an important role in maintaining the levels of SPi-B and IRF4 in ABC-DLBCL [39]. Following CRBN downregulation, the mRNA and protein levels of SPi-B and IRF4 were reduced [39]. SPi-B is required for ABC-DLBCL cells survival and its knockdown may have toxic effects on ABC-DLBCL cells [54]. Similarly, IRF4 expression is regarded as a hallmark of ABC DLBCL and its overexpression confers ABC-DLBCL cells resistant to lenalidomide [29, 39]. Spi-B together with IRF4 reduces the expression of INF-*β* and influences the survival and proliferation of cells in ABC-DLBCL [55]. There may be a positive interaction mechanism between IRF4 and NF-*κ*B, because NF-*κ*B enhances IRF4 transcription and IRF4 enhance NF-*κ*B activity. The downregulation of IRF4 inhibits NF-*κ*B activity, and conversely, the overexpression of IRF4 enhances NF-*κ*B activity and results in lenalidomide resistance in ABC-DLBCL cells [53].

Research indicates that treatment with lenalidomide significantly inhibited CLL cells proliferation, which is associated with the p53-independent upregulation of a cyclin-dependent kinase inhibitor p21. Silencing of CRBN impaired the effect of lenalidomide to induce p21 expression as well as CLL cell proliferation. These results indicate that lenalidomide directly inhibits the CLL cell proliferation in a CRBN/p21-dependent manner [56].

The CSNK1A1 gene, whose expression product is CK1*α*, located in the common deleted region for del (5q) MDS [57]. CK1*α* has tumor-suppressing capabilities and is closely related to the biological and therapeutic effect of del (5q) MDS [57, 58]. Lenalidomide application reduces its expression [59]. However, no similar phenomenon was observed when cells treated with the proteasome inhibitor or other nonimmunomodulatory drugs. The lenalidomide-dependent decrease in CK1*α* protein level was induced by the ubiquitin action of the CRBN-related E3 ubiquitin ligase complex. It was shown that the lenalidomide-induced ubiquitination of endogenous CK1*α* only occurred in the presence of CRBN [60]. Last year, Fang et al. identified a candidate gene GPR68, whose expression products has been implicated in calcium metabolism, for modulating the sensitivity to LEN in MDS cells. They found lenalidomide-induced GPR68 expression via IKZF1, resulting in the increasing level of cytosolic calcium and activating a calcium-dependent calpain CAPN1, which plays an important role in the induction of apoptosis of MDS cells. Depletion of calpastatin, an endogenous CAPN1 inhibitor which is encoded by a gene deleted in del(5q) MDS, increased the expression of CAPN1 and enhanced the sensitivity of del(5q) MDS to lenalidomide [61]. Taken together, these studies indicate the essential role of CRBN in the treatment of del(5q) MDS and provide an explanation for the superior responses of patients with del(5q) MDS to lenalidomide treatment.

### 3.5. Novel Agents and Newly Discovered Signal Pathways of CRBN

Many researchers have sought to identify new CRBN agents and new signal pathways of CRBN, which may allow a wider range of application in treating more diseases. Matyskiela et al. reported a novel CRBN agent CC-885 with potent ability to inhibit cancer cell line growth as well as patient-derived acute myeloid leukemia cells. CC-885 promoted ubiquitination and degradation of translation termination factor GSPT1 (eRF3a) [62]. GSPT1, cooperatively with eRF1, induces effective stop codon recognition [63]. The degradation of GSPT1 leads to cell cycle arrest thereby ensuring the antitumor effect of CC-885 [62].

CRBN also negatively regulates the Toll-like receptor 4- (TLR4-) mediated signal pathway, thereby downregulating NF-*κ*B expression and proinflammatory cytokine production [64]. CRBN binds to the zinc finger domain of tumor necrosis factor (TNF) receptor-associated factor 6 (TRAF6), which contains an autoubiquitination site [65]. The CRBN-TRAF6 interaction attenuates the ubiquitination of TRAF6 and TAB2, thereby inhibiting the activity of NF-*κ*B and the production of proinflammatory cytokines [65]. *In vivo* experiments showed that CRBN knockdown mice were more vulnerable to lipopolysaccharide (LPS) challenge and that this effect might be associated with enhanced NF-*κ*B activity and increased proinflammatory cytokines [64].

## 4. Conclusions

CRBN is closely associated with the proliferation and metabolism of normal cells as well as tumor cells. Its existence ensures normal metabolic function and normal physiological function of ion channels, thereby maintaining cell growth and proliferation. CRBN is also involved in the occurrence of many diseases. In addition, with the identification of CRBN and its multiple functions, the antitumor action and the relative side effects of IMiDs have become more understandable. Evidence indicates that CRBN is an indispensable protein for IMiDs function. Furthermore, CRBN is a direct target of IMiDs and is related to the sensitivity and response to IMiDs, providing a theoretical foundation for individualized clinical therapy.

However, the mechanisms involved in CRBN functions are still poorly understood. What is the underlying mechanism through which cellular contents of CRBN can be decreased after long-term treatment with IMiDs in MM patients or MM cells? What is the intrinsic mechanism of the different CRBN protein levels among different cell lines sensitive or resistant to IMiDs? If eliminating CRBN expression can suppress cell proliferation and induce apoptosis, then why is the viability of cells that express low levels of CRBN protein similar to cells that highly express CRBN protein? Many animal experiments have shown that the inhibition of CRBN reduces the genesis of cardiovascular disease and obesity: could this be the same in humans? The development of drugs that combine with IMiDs to overcome IMiDs resistance resulting from the IMiD-mediated downregulation of CRBN might provide better combined clinical treatment.
